# Delayed diagnoses of mitochondrial cytopathies in patients presenting with end stage kidney disease: two case reports

**DOI:** 10.1186/s12882-020-02002-5

**Published:** 2020-08-24

**Authors:** Tayeba Roper, Mark Harber, Gareth Jones, Robert D. S. Pitceathly, Alan D. Salama

**Affiliations:** 1 Department of Renal Medicine, Guy’s & St Thomas’ NHS Foundation Trust, Great Maze Pond, London, UK; 2 UCL Department of Renal Medicine, Royal Free Hospital, Pond Street, Hampstead, London, UK; 3 Department of Neuromuscular Diseases, UCL Queen Square Institute of Neurology and The National Hospital for Neurology and Neurosurgery, London, UK

**Keywords:** End stage renal disease, Mitochondrial cytopathies, Primary mitochondrial disease, Renal transplant, Delayed diagnoses, Inherited conditions, Multisystem disorders

## Abstract

**Background:**

Up to one third of patients on renal replacement programmes have an unknown cause of kidney disease, and the diagnosis may only be established following renal transplantation when the disease recurs or if new extra-renal symptoms develop.

**Case presentation:**

We present two patients who presented with progressive chronic kidney disease of unknown cause. Both patients underwent successful renal transplantation but subsequently developed multisystem abnormalities, and were ultimately diagnosed with mitochondrial cytopathy 10–15 years following transplantation.

**Conclusions:**

Mitochondrial cytopathies are rare inborn errors of metabolism that should be considered in adults with renal impairment, especially in those with a family history of kidney or other multisystem disease. The widespread availability of genetic testing provides the potential for earlier diagnoses, thereby enhancing management decisions, anticipation of complications, avoidance of mitotoxic drugs, and informed prognosis prediction.

## Background

Mitochondrial cytopathies (MC), also known as primary mitochondrial diseases (PMD), are a rare heterogenous group of conditions, defined by sporadic or inherited mutations in either mitochondrial DNA (mtDNA) or nuclear DNA (nDNA), that encode for proteins required for mitochondrial function [1]. Consequently, MC may follow maternal or Mendelian inheritance patterns, depending on the location of the mutant gene [2]. During mitosis the distribution of mtDNA to daughter cells is uneven, and so cells often contain a mixture of both mutant and wild-type mtDNA [3]. Cells are therefore either homo- or heteroplasmic with regard to mutations, i.e. contain entirely mutant mtDNA, or a varying amount of both wild-type and mutant mtDNA, respectively [4]. The overall frequency of mtDNA mutations in adults is estimated to be 1:5000 [5]. MC are often multisystem disorders and frequently affect organs or tissues with high metabolic demand, such as the brain, heart and skeletal muscle [4, 6]. MC can present as Mitochondrial Cytopathy Syndromes (MCS) affecting multiple organs, with certain clinical features occurring in clusters. Several different MCS have been reported (Table 1), however the majority of MC do not fall within these clinical entities [10]. Furthermore, different genotypes can present with the same phenotype (Table 1), which may be related to different penetrance associated with mtDNA heteroplasmy.

**Table 1:** Summary of mitochondrial cytopathy syndromes frequently associated with renal phenotypes

MCS	Clinical Characteristics	Renal Phenotype	Genotype	Reference
**Kearns-Sayre Syndrome (KSS)**	Progressive external ophthalmoplegia, Retinal Pigmentary degeneration, Progressive myopathy, Cerebellar ataxia, Cardiomyopathy, Heart block	Barter-like syndrome, RTA, Fanconi syndrome, Severe tubulopathy	Single large-scale mtDNA deletions	[1, 6]
**Mitochondrial Encephalopathy, Lactic acidosis and Stroke-like** **Episodes (MELAS)**	Stroke-like episodes (hemiparesis, hemianopia, cortical blindness), Epilepsy, Dementia, Lactic acidaemia, Recurrent headaches, Diabetes, Sensorineural hearing loss, Short stature	FSGS, TIN	Mutations in mtDNA, most commonly m.3243A>G	[2, 7, 8]
**Myoclonus Epilepsy and Ragged Red Fibres (MERRF)**	Myoclonus, Epilepsy, Cerebellar ataxia, Sensorineural hearing loss, Myopathy, Optic atrophy, Short stature, Dementia Muscle biopsy – ragged red fibres	FSGS, Chronic TIN, Cystic renal disease (1 case)	Mutations in mtDNA, most commonly m.8344A>G	[2, 9]
**Leber Hereditary Optic Neuropathy (LHON)**	Visual loss with optic atrophy, Wolff-Parkinson-White syndrome, Multiple sclerosis-like disease	TIN	Mutations in mtDNA, most commonly m.11778G>A, m.3460G>A, m.14484T>C	[6, 10–12]
**Maternally Inherited Diabetes & Deafness (MIDD)**	Sensorineural hearing loss, Diabetes, Macular retinal dystrophy, Myopathy, Short stature, Gastrointestinal disease	FSGS	Point mutations in mtDNA, most commonly m.3243A>G	[13]
**Leigh Disease**	Developmental delay, Ataxia, Dementia, Dystonia, Seizures, Vomiting, Respiratory failure	Fanconi syndrome	Mutations in mtDNA and nDNA, most commonly involvi\ng complex I genes	[1]
**Pearson Syndrome**	Severe anaemia, Neutropenia, Sensorineural hearing loss, Thrombocytopenia, Exocrine pancreatic insufficiency Bone marrow biopsy – ring sideroblasts	Tubulopathy, FSGS, Crescentic GN, Mesangial proliferation	Single large-scale mtDNA deletions	[7]
**COQ10 Biosynthesis Defects**	Cerebellar Ataxia, Isolated myopathy, Encephalopathy, Myoglobinuria, Sensorineural hearing loss	Nephrotic syndrome (FSGS), Tubulopathy	Mutations in 8 nuclear encoded mitochondrial genes; PDSS1/2, COQ2/4/6/9, ADCK3/4	[2, 14]

MC have increasingly been recognised to cause renal disease. This is unsurprising, given the high level of metabolic activity within the kidneys [4, 7]. The majority of MC with renal manifestations present in childhood, but can also present in adults [1]. Diagnosing MC can be challenging, given their heterogeneity, but should be considered when multiple systems are involved or if there is a positive family history [3, 10]. Renal histology may show the presence of dysmorphic mitochondria on electron microscopy [2, 3]. However, MC are generally diagnosed through measurement of mitochondrial oxidative phosphorylation enzyme activities, alongside genetic testing. In the case of mtDNA, analysis of multiple tissue types, including blood leucocytes, urinary epithelial cells and skeletal muscle tissue, is often required [2, 6]. Here, we report two patients presenting with renal disease in whom the diagnosis of MC was made several years after their initial presentations with end stage renal disease (ESRD), following the development of progressive, non-renal, multisystem manifestations of mitochondrial dysfunction.

## Case presentations

### Case 1

A 22-year-old woman was referred to her local renal unit for assessment of hypertension and advanced renal impairment, with a serum creatinine of 500 μmol/l. She had a normal birth and childhood development. Her medical history was unremarkable, except for sensorineural deafness, requiring hearing aids. There was no family history of kidney disease. Investigations revealed a negative autoimmune screen, including a negative ANCA. She proceeded to a renal biopsy, which demonstrated diffuse segmental sclerosis in all glomeruli, marked tubular atrophy, interstitial fibrosis and chronic inflammation. Small vessels were constricted with some fibrinoid change in the walls. This was interpreted as being due to a pauci-immune ANCA-negative vasculitis. She received 3 months of cyclophosphamide and prednisolone, followed by azathioprine and prednisolone.

Following a rapid and progressive decline in kidney function, she was established on peritoneal dialysis. Two years later she underwent a living unrelated donor renal transplant, achieving excellent renal function with creatinine between 90 and 100 μmol/l within 2 weeks after transplantation. After 1 year she developed post transplantation diabetes mellitus (PTDM) and was commenced on long acting insulin, with subsequent addition of metformin. She later developed amenorrhea and dizziness.

**Fig. 1:**
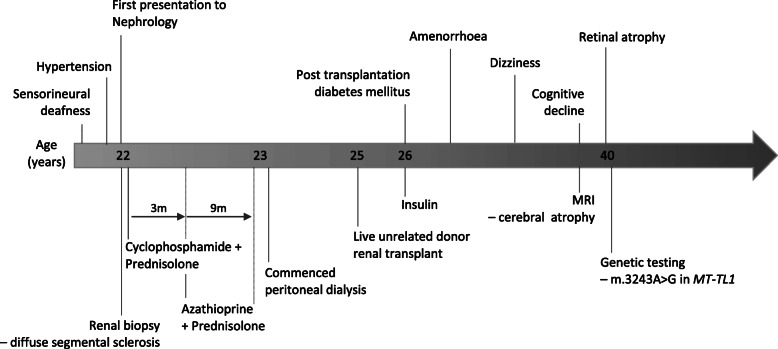
Timeline of events Case 1

Fifteen years post renal transplantation she was assessed by an ophthalmologist, following a deterioration in vision, who diagnosed retinal atrophy. She subsequently developed cognitive decline associated with cerebral atrophy on brain MRI. As a result of the neurological symptoms and signs, and in view of the retinal abnormalities, further genetic testing was undertaken. Restriction fragment length polymorphism of mtDNA extracted from blood leukocytes and urinary epithelial cells confirmed the m.3243A > G mutation in *MT-TL1* at 21 and 14% mutant loads, respectively. Maternal screening was declined. The patient was commenced on Co-enzyme Q10 supplementation. Renal function has remained stable with creatinine between 75 and 90 μmol/l during the 22 years following post transplantation. She had no vasculitis related illness throughout her prolonged follow up, suggesting the MC was the likely cause of her ESRD. Case 1 is summarised in Fig. 1.

### Case 2

A 31-year-old woman with a background of Asperger’s syndrome, thyroid goitre, aortic and mitral valve regurgitation, and sensorineural deafness, presented with ESRD. There was no family history of renal disease. At the time of presentation an acute renal immunology screen was negative and ultrasound scan demonstrated bilateral small, smooth kidneys. Renal biopsy was therefore not possible and she was commenced on haemodialysis. Five months later she received a living-related donor renal transplant from her mother. Graft function following transplantation was excellent, with a creatinine that reached a nadir of 88 μmol/l after 4 weeks and stabilised over a 10 year period at 110 μmol/l.

**Fig. 2:**
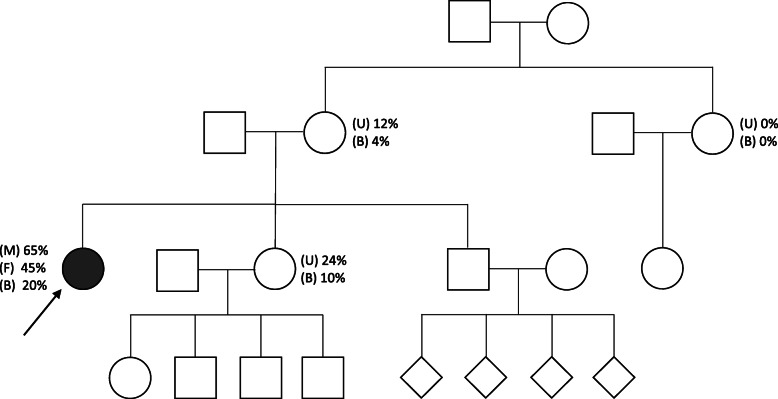
Family pedigree structure Case 2. Percentage heteroplasmy in muscle (M), fibroblasts (F), blood (B) and urine (U) are shown (adapted from [15])

**Fig. 3:**
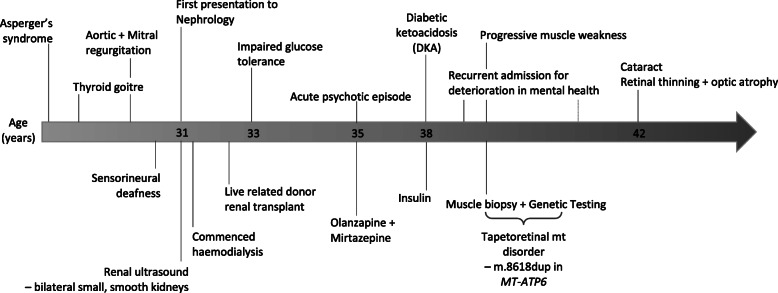
Timeline of events Case 2

Two years following transplantation the patient developed impaired glucose tolerance, treated with dietary modification. Three years later she developed diabetic ketoacidosis, requiring hospital admission, and was commenced on insulin therapy. Four years post transplantation she was hospitalised with an acute psychotic episode for which she commenced Olanzapine and Mirtazepine. A number of admissions followed with deterioration in mental health. Six years following the transplant there was a gradual reduction in mobility and she was investigated by neurologists who performed a muscle biopsy and genetic testing. Muscle histology revealed mild myopathic changes with no specific features of mitochondrial disease. Next generation sequencing of mtDNA extracted from blood leukocytes, cultured fibroblasts and skeletal muscle tissue confirmed the presence of the pathogenic m.8618dup in *MT-ATP6* at 20, 45 and 65% mutant loads, respectively [15]. The mutation was detected in other maternal relatives, albeit at lower levels (Fig. 2). She has since developed cataracts, which required removal, retinal thinning and optic atrophy, but no evidence of diabetic retinopathy. Case 2 is summarised in Fig. 3, and previously reported in [15].

## Discussion and conclusions

Renal complications of MC are uncommon, but can present in several ways. The most frequent renal manifestation in children is tubulointerstitial nephritis (TIN) [11], while in adults the presence of proximal tubular dysfunction is more common [1]. Tubular defects range from mild tubular disease (e.g. isolated hypomagnesiaemia, hypercalcuria or renal tubular acidosis) to a complete Fanconi syndrome [2]. However, tubular defects may be overlooked in the context of other, more severe, organ involvement [7, 16]. Glomerular disease has also been reported, generally with focal segmental glomerulosclerosis (FSGS) and associated proteinuria. Rarely, MC have been found to cause chronic TIN and cystic renal disease [2]. In both cases presented, the exact mechanism of renal injury remains unknown, due to the advanced stage of renal impairment at the time of presentation. The late onset presentation of renal disease in these two patients, might have been a result of genetic drift of mtDNA heteroplasmy over time. In rapidly dividing cells, such as blood leukocytes, there is a reduction in mutant mtDNA heteroplasmy over time, while the opposite is thought to occur in post-mitotic cells [17]. A clinical phenotype is only observed when the mutant mtDNA load exceeds a mutation specific biochemical ‘threshold’ [2, 3]. Interestingly, Case 2 exhibited a lower mutant load in urinary epithelial cells than blood, despite the former usually correlating more closely with post-mitotic tissues, such as muscle tissue. It is possible the donor renal transplant contributed towards the lower than expected level of mutant mtDNA, compared to native kidneys and bladder derived cells. However, further genetic testing of urinary epithelial cells pre- and post-transplant would be required to investigate this finding further.

As demonstrated in both cases, the vast array of affected organ systems and different clinical features of MC and MCS presenting over a protracted time period, make diagnosis of these conditions a challenge. This is further complicated by the fact that many routine investigations for MC can present with variable, including normal, findings even in the presence of disease. Specific routine investigations often include biochemical tests to quantify creatine kinase, blood glucose, serum lactate and pyruvate. These can all be raised in MC, however levels can vary with different phenotypes and may be normal in some. Skeletal muscle biopsy was previously the gold standard investigation for identifying MC. Modified Gomori trichrome staining can reveal the abnormal accumulation of sub-sarcolemmal mitochondria, giving the typical ‘Ragged Red Fibre’ appearance on microscopy. Electron microscopy can be used to visualise mitochondria and determine morphology, presence of abnormal inclusions and pleomorphic mitochondria. Furthermore, analysis of enzyme activity from biopsy specimens can identify specific deficiencies of complexes of the respiratory chain [18]. As with biochemical tests, and as demonstrated in Case 2, muscle biopsy results can also be normal even in the presence of disease. Case 1 highlights how many MC are now diagnosed without the need for muscle biopsy. Following advances in next-generation sequencing, genetic analysis of nDNA extracted from blood, or mtDNA extracted from blood leukocytes, urine epithelial cells or affected tissues, can identify mutations responsible for MC [19]. Many commercial kits are now available to identify known mutations associated with MC, alternatively whole genome sequencing can be used to identify previously unknown mutations [20].

Management of MC requires a multidisciplinary team approach, and often involves supportive care alongside targeted treatment of organ-specific complications (e.g. renal replacement therapy for ESRD) [21]. Disease-specific treatments are also available for certain MCS, and often involve replacement of specific components involved in mitochondrial function. Coenzyme Q10 is one such component of the respiratory chain. Supplementation with Coenzyme Q10, as in Case 1, and more recently Coenzyme Q10 analogues (e.g. Idebenone), is often used in patients with a MC which may cause a deficiency of this enzyme, and can result in a delay in the progression of disease. However, timely initiation of therapy is key in order to observe any benefit. Similarly, supplementation with other antioxidants or cofactors involved in the respiratory chain, such as; L-arginine, L-carnitine, thiamine, creatine phosphate and vitamins B2, C and E, may also provide some clinical benefit [22]. In contrast, confirming a diagnosis of a MC can also allow for appropriate avoidance of medications which may exacerbate disease. These include certain anaesthetic agents (e.g. propofol) which may depress mitochondrial function, substances metabolised by mitochondria (e.g. sodium valproate), inhibitors of the respiratory chain (e.g. metformin, statins) and mitochondrial protein synthesis inhibitors (e.g. aminoglycosides, tetracyclines) [20]. Overall management of these conditions remains largely supportive. Further advances in therapies continue to be investigated, and include the use of gene therapy, enzyme replacement and methods to stimulate mitochondrial proliferation and alter heteroplasmy [20, 22]. In both the cases, earlier diagnosis may have allowed for more timely initiation of appropriate management, including avoidance of medications such as metformin. Although it will remain unknown, this could have potentially impacted on the development of extra-renal disease manifestations.

The cases presented are typical of many patients with an underlying MC, in that they develop disorders of multiple organ systems over time, with no specific symptoms pointing towards an underlying common cause. Diabetes is one such feature which is found to be present in many patients with MC, particularly those with m.3243A > G mutations. The clinical picture was complicated in both cases, as both were thought to have developed PTDM. PTDM is common, occurring in up to one third of non-diabetic patients following transplantation [23]. Many studies have identified single nucleotide polymorphisms, previously linked to both type 1 and type 2 diabetes mellitus, to be associated with an increased risk of developing PTDM, none of these however are associated with mtDNA or mitochondrial function [24]. Studies have shown that, although many patients with MC develop diabetes in childhood, the heteroplasmic nature of the mitochondrial mutation within cells means diabetes can also develop later in life. Such patients have been shown to develop progressive loss of insulin secretion from pancreatic β-cells in response to glucose stimulation, as opposed to insulin resistance at the cellular level [25]. As a result, these patients often become insulin-dependent. The use of Metformin should be avoided due to the risk of developing severe lactic acidosis, as metformin is known to inhibit components of the respiratory chain [25]. It is possible that in both cases presented the development of diabetes was related purely to the underlying MC. Alternatively it could be that there was a low level of heteroplasmy within the pancreatic β-cells of these patients, but with the addition of immunosuppressant medication (steroids and calcineurin inhibitors) post-transplantation, they were tipped into a diabetic phenotype.

Up to as many as 28% of patients established on renal replacement therapy (RRT) have no identified cause of ESRD [26, 27]. Furthermore, many more patients, have a presumed diagnosis made with no biopsy evidence of the underlying pathology. As demonstrated by these two cases, diagnosing MC can be a challenge. In Case 1, the correct diagnosis was not initially made, despite renal biopsy, as this showed non-specific features of diffuse segmental sclerosis with interstitial fibrosis and tubular atrophy. The presence of segmental sclerosing lesions on renal biopsy is a common feature observed in MC but can also be seen secondary to a host of other aetiologies [28, 29]. As discussed above, further diagnostic delay occurred in both patients, as the PTDM was presumed to be exclusively due to immunosuppressive therapy, and not the common presenting feature of many MCS [30]. It was not until both patients developed progressive neurological features that an all-encompassing, alternative diagnosis was considered and investigated. Clinicians should consider a possible underlying diagnosis of a MC particularly in patients with no identified cause of ESRD, who develop diabetes and have symptoms relating to two or more other organ systems. Suspicions of an underlying MC may be further heightened in those with a strong family history of renal disease and/or diabetes, and in those with symptoms suggestive of an underlying muscle or neurological disorder [19].

Although relatively rare, MC are increasingly recognised as a cause of renal impairment and ESRD. MC should be considered when assessing patients presenting with renal disease of unknown aetiology, particularly if a family history of renal impairment exists, and/or if there is evidence or development of multi-system disease. Normal investigation results do not necessarily exclude a diagnosis of a MC. Consequently, if there is a high degree of clinical suspicion, referral to a specialist centre for further assessment and genetic testing should be considered.

## Data Availability

The datasets generated and/or analysed during the current study are available in the NCBI dbsnp repository, https://www.ncbi.nlm.nih.gov/clinvar/variation/9589/ and https://www.ncbi.nlm.nih.gov/clinvar/variation/9648/.

## Abbreviations

ESRD: End stage renal disease
FSGS: Focal segmental glomerulosclerosis
MC: Mitochondrial cytopathies
MCS: Mitochondrial cytopathy syndromes
mtDNA: Mitochondrial deoxyribonucleic acid
nDNA: Nuclear deoxyribonucleic acid
PMD: Primary mitochondrial diseases
PTDM: Post transplantation diabetes mellitus
RRT: Renal replacement therapy
TIN: Tubulointerstitial nephritis
tRNA: Transfer ribonucleic acid
